# Cubogel as potential platform for glaucoma management

**DOI:** 10.1080/10717544.2021.1872740

**Published:** 2021-01-29

**Authors:** Sinar Sayed, Mostafa Abdel-Moteleb, Maha Mohamed Amin, Omnia Mohamed Khowessah

**Affiliations:** a Pharmaceutics and Industrial Pharmacy, Cairo University Faculty of Pharmacy, Cairo, Egypt; b Quality Assurance Department, Sigma Tec Pharmaceutical Company, Giza, Egypt

**Keywords:** Cubogel, glaucoma, terminal sterilization, IOP, stability and corneal bioavailability

## Abstract

The aim of this work is to survey the potential of cubogel as an ocular dosage form to boost the corneal permeability of Dorzolamide Hydrochloride DZ; an antiglaucomal drug. DZ-loaded cubosomal dispersions were prepared according to Box-Behnken design, where the effect of independent variables; Monoolein MO concentration (2.5, 5 and 7.5%w/w), Pluronic^®^ F127 concentration (0.25, 0.5 and 0.75%w/w) and magnetic stirrer speed of (400, 800 and 1200 rpm) was studied on PS (nm), Zp (−mV) and Q 2 h (%) respectively. The prepared formulae were characterized via drug content DC (%), particle size PS (nm), polydispersity index PDI, zeta potential Zp (−mV), *in-vitro* drug release (Q 2 h%) and finally TEM. The optimized formulation composed of: 6.13% w/w of MO, 0.75% w/w of F127 and prepared at 1200 rpm stirring speed was chosen based on the criteria of minimum PS (nm), maximum Zp (−mV) and minimum Q 2 h (%). Results revealed that the optimum formula showed PS of 153.3 ± 8.4 n, Zp of 32 ± 3 −mV and 37.78 ± 1.3% released after 2 h. Carbopol 934 (1% w/v) as gelling agent was used to prepare the optimum cubogel, which was further evaluated by DSC, *ex-vivo* permeation and stability studies at 4 °C for three months. Moreover, *in vivo* studies of the optimized cubogel include; draize test, histological examination, confocal laser scanning microscopy (CLSM) and intraocular pressure (IOP) measurement. Results revealed that the optimized cubogel was considerably safe, stable and competent to corneal delivery as assured by draize and histological examination. CLSM showed a deeper penetration of more than 2.5-fold. A higher bioavailability (288.24 mg. h/ml) was attained from cubogel compared to the market product Trusopt^®^ eye drops (115.40 mg. h/ml) following IOP measurement. Therefore, DZ-loaded cubogel could be considered as promising delivery system to boost the transcorneal permeation hence corneal bioavailability of DZ as antiglaucomal drug.

## Introduction

Cubosomes are thermodynamically stable self-assembled nanoparticles cubic liquid crystalline phases. Cubosomes have many advantages including; capability to incorporate many hydrophilic, lipophilic and amphilic drugs, ease of preparation adopting simple techniques and economics, inclusion of biodegradable lipids which forming nanovesicles with enhanced permeation power, protection of the encapsulated drug from physical and chemical degradation, controlled target drug delivery system and capability to load great amount of drug owing to the cubic structure with high stability and biocompatibility (Gaballa et al., 2019).

Being thermodynamically stable, cubosomes can be simply merged into drug formulations due to their ability to remain stable almost at any dilution level as a result of insolubility of the cubic structure which form lipid in water, this cubic structures of cubosomes can be ruptured and diffused to form particulate diffusions that are colloidally and thermodynamically stable for prolonged time (He et al., 2017; Rao et al., 2018; Gaballa et al., 2019).

Cubosomes drug delivery had been widely used in management of several diseases with different routes. Cubogels could be considered as challenge carriers for brain targeting through intranasal route and promising in oral delivery of several drugs suffering from low aqueous solubility, poor absorption, and large molecular size. Cubosomes as versatile drug nano carriers can be targeted via transdermal route. Also it could be considered as a novel dosage form for the treatment of epileptic children and also show an innovative advance in gene therapy. Subsequently, cubosomes as a novel drug delivery system could be a challenge for advanced and targeted drug delivery systems (Karami & Hamidi, 2016).

Ocular drug delivery faces various challenges, as low corneal permeability and bioavailability as a result of the characteristic anatomical structure of the human eye (Rao et al., 2018). Cubosomes showed highly promising for ocular disease treatment such as glaucoma with the advantage of increasing drug corneal permeability and bioavailability (Rao et al., 2018). Glaucoma is a chronic ophthalmic disease caused by degeneration of neuronal tissue as a result of different eye diseases that in majority of patients causes increased pressure within the eye (Boia et al., 2020). This high pressure is triggered by backing of fluid in the eye that by long time produces optic nerve damage. Only early diagnosis, discovery and healing can aid to reserve vision in glaucoma. Symptoms and signs of glaucoma are; Increase intraocular pressure (IOP), discomfort, obstructed veins and swelling of the eye (Öhnell et al., 2019).

Treatment of glaucoma can be achieved by lowering IOP either via medical therapy or eye surgery (Sheybani et al., 2020). Most antiglaucomal drugs are classified pharmacologically into adrenergic agonists as Brimonidine, adrenergic antagonists as Timolol, carbonic anhydrase inhibitors as Dorzolamide, cholinergic agents as Pilocarpine and derived prostaglandins as Latanoprost which are considered lifelong medications in order to lower ocular hypertension and keep IOP at an acceptable level (Yu-Jie et al., 2020) Dorzolamide hydrochloride as carbonic anhydrase inhibitor is one of the first medical therapy line of glaucoma. Suppression of carbonic anhydrase in the ciliary functions of the eye will thereby reduce aqueous humor discharge apparently by retarding the development of bicarbonate ions with consequent lowering in sodium and fluid transportation (Sayed et al., 2020).

Different techniques were used to improve and sustain the ocular bioavailability of DZ. Many DZ-loaded nanoparticle drug delivery systems were successfully prepared; proniosomal gels (Fouda et al., 2018; Sayed et al., 2020) and nanostructured carriers (Afify et al., 2018) showed great improvement in the pharmacokinetic parameters and extend the drug pharmacological action. DZ-loaded in-situ chitosan nanoparticles (Katiyar et al., 2014), nanoliposomes (Kouchak et al., 2018), self-aggregating nanoparticles (Gudmundsdottir et al., 2014) and chitosan-coated polycaprolactone nanoparticles loaded with DZ (Shahab et al., 2020) were also formulated.

The aim of this study is to develop a novel DZ-loaded cubogel as an ocular drug delivery system with prolonged ocular residence time, enhanced corneal permeation, sustained drug release with prolonged IOP lowering effect over 12 h, thereby enhancing DZ ocular bioavailability, reducing frequency of administration resulting in higher patient compliance.

## Materials and methods

### Materials

Dorzolamide-HCl was a gift from (Epico-pharmaceutical Co., Cairo, Egypt). DL-α-Monoolein (MO), Tween 80^®^ (T80), Pluronic ^®^ F127 (F127) and Rhodamine B (RhB) were purchased from (Sigma-Aldrich^®^ Inc, USA). Carbopol 934 was obtained from (Goodrich chemical company, Charlotte, UK). Ethyl alcohol 95%, Methyl alcohol, Disodium hydrogen phosphate, Potassium dihydrogen phosphate and Sodium chloride were purchased from (El Gomhoria., Kasr El-Aini street, Egypt). All used water was deionized distilled water.

### Statistical design of the study

Box-Behnken design from Design-Expert^®^ software (Version 7, Stat-Ease Inc., MN, USA) was carried out to study the effect of different formulation variables on the prepared cubosomal dispersion. As shown in Table 1 the main independent variables; A: MO (range between 2.5 and 7.5%w/w), B: F127 (range between 0.25 and 0.75%w/w) and C: Speed of magnetic stirrer (range between 400 and 1200 rpm), with the corresponding dependent variables; Y1: PS (nm), Y2: Zp (−mV) and Y3: *In-vitro* release after two hours (Q 2 h) (%) respectively. Table 2 shows the different prepared DZ-loaded cubosomal dispersion formulae and their measured responses (Abdelrahman et al., 2015).

**Table 1.** Box–Behnken Design showing both independent and dependent variables with their respective constraints used for the preparation of DZ-loaded cubosomal dispersion formulae.

Factors	Levels			Responses	Constraints
(Independent variables)	Low (−1)	Medium (0)	High (+1)	(Dependent variables)	
A: MO conc (% w/w)	2.5	5	7.5	Y1: PS (nm)	Minimize
B: F127 conc (%w/w)	0.25	0.5	0.75	Y2: Zp (− mV)	Maximize
C: Speed of stirring (rpm)	400	800	1200	Y3: Q2 h (%)	Minimize

**Table 2.** Box–Behnken Design of the different prepared DZ-loaded cubosomal dispersion formulae and their measured responses.

	Factors levels							
Formulation	A:MO concentration (% w/w)	B: F127 concentration (% w/w)	C: Speed of magnetic (RPM)					
				DC (% ± SD)	PS (nm ± SD)	PDI (± SD)	*Zp* (−mV ± SD)	Q 2h (% ± SD)
F1	2.5	0.25	800	91.65 ± 1.95	554.25 ± 128.25	0.680 ± 0.013	31.90 ± 1.80	64.73 ± 1.13
F2	7.5	0.25	800	92.78 ± 1.76	583.60 ± 160.90	0.587 ± 0.029	36.40 ± 0.50	67.86 ± 1.02
F3	2.5	0.75	800	92.34 ± 2.58	306.55 ± 078.95	0.352 ± 0.017	33.55 ± 1.65	54.49 ± 0.49
F4	7.5	0.75	800	91.11 ± 1.09	384.50 ± 140.50	0.576 ± 0.148	34.00 ± 1.70	56.13 ± 1.65
F5	5	0.5	800	90.90 ± 0.50	576.44 ± 45.40	0.856 ± 0.035	24.2 ± 1.9	59.94 ± 1.2
F6	2.5	0.5	400	93.83 ± 1.63	643.80 ± 206.50	0.513 ± 0.032	35.00 ± 0.40	75.57 ± 0.65
F7	7.5	0.5	400	95.70 ± 1.50	714.20 ± 143.80	0.667 ± 0.213	34.50 ± 0.90	74.43 ± 0.48
F8	2.5	0.5	1200	96.07 ± 0.61	206.35 ± 2.75	0.344 ± 0.017	32.90 ± 0.30	52.60 ± 0.21
F9	7.5	0.5	1200	94.76 ± 0.96	195.05 ± 0.25	0.340 ± 0.027	35.95 ± 1.65	38.36 ± 0.99
F10	5	0.25	400	92.48 ± 1.08	833.45 ± 169.55	0.628 ± 0.174	34.20 ± 0.20	75.67 ± 0.75
F11	5	0.75	400	93.61 ± 1.14	599.10 ± 155	0.612 ± 0.114	34.85 ± 1.95	72.15 ± 1.04
F12	5	0.5	800	91.10 ± 0.40	541.72 ± 89.72	0.518 ± 0.0165	30.1 ± 1.8	62.31 ± 0.91
F13	5	0.25	1200	92.41 ± 2.49	174.50 ± 4.50	0.352 ± 0.029	37.45 ± 1.05	30.14 ± 1.04
F14	5	0.75	1200	98.02 ± 0.42	161.55 ± 0.25	0.192 ± 0.010	37.20 ± 0.40	25.64 ± 0.33
F15	5	0.5	800	90.50 ± 0.60	559.07 ± 17.37	0.759 ± 0.241	27.15 ± 2.95	61.12 ± 1.18

All formulae contain 2% w/w of DZ and 0.5% w/v of T80.

### Preparation of DZ-loaded cubosomal dispersion formulae

Cubosomal nanodispersions were prepared adopting ‘Melt dispersion-emulsification’ method in which the melted lipid phase containing the specified amount of MO and F 127 using hot magnetic stirrer kept at 60 ± 2 °C (Jenway, United Kingdom). A specified amount of DZ was mixed with the melted lipid portion with continuous stirring (Esposito et al., 2003; Eldeeb et al., 2019; Hakeem et al., 2020). The mixture of lipid phase and DZ was dispersed into pre-heated aqueous phase containing T80 solution using magnetic stirring for 1 h (Carr et al., 1973). Finally, the cubosomal dispersion was obtained and stored at room temperature for more investigations.

### Characterization of the prepared DZ-loaded cubosomal dispersions

#### Drug content DC (%)

A certain amount of the prepared cubosomal dispersion was calculated, where 0.2 ml (equivalent to 4 mg DZ) of each formulation was mixed with 10 ml methanol, then filtered with 0.45 µm membrane filter (sigma Aldrich) to remove any unsolubilized excipient and measured spectrophotometrically (Shmiadzu type (UV-1800-240v), Corp, Japan) at *λ*_max_ of 253 nm against methanol as blank (Esposito et al., 2005; Hundekar et al., 2014) and drug content percentage DC (%) was calculated as follows:

$$\text{DC }\left(\%\right)=\frac{\text{Actual yield}}{\text{Theoretical yield}}\times 100$$

#### Particle size PS (nm) and polydispersity index PDI

The mean PS (nm) and PDI of the different DZ-loaded prepared cubosomal dispersions were detected using Zeta Seizer (Malvern Instrument Ltd., Worcestshire, UK), within a detection limit of 0.1–2000 µm. All samples were prepared by dilution (100 times) of cubosomal dispersion with deionized water before measurement (Jin et al., 2013; Matloub et al., 2018).

#### Zeta potential Zp (-mV)

Zeta potential Zp (−mV) values of the different prepared DZ-loaded cubosomal dispersions were also detected via laser Doppler anemometry of Zetasizer instrument (Malvern Instruments, Malvern, UK) at a temperature of 25 °C (Matloub et al., 2018).

#### *In-vitro* release (Q 2 h)

*In-vitro* release of DZ from the prepared cubosomal dispersion formulae were determined using pretreated dialysis bag (typical molecular weight cutoff 14,000 Da; Sigma-Aldrich Co) as a donor compartment (Aggarwal & Kaur, 2005). One ml of phosphate buffer saline (PBS pH 7.4) was added to a certain volume of cubosomal dispersion equivalent to 5 mg DZ, in a dialysis bag, stacked from both sides with closures, and placed in a beaker containing 50 ml of PBS pH 7.4 as receptor compartment. The beaker was placed in a shaker (GFL, Gesellschatt laboratories, Berlin, Germany) with agitation speed of 50 rpm maintained at 32.5 ± 0.5 °C. The withdrawn samples at the specified time intervals (0.5, 1, 2, 3, 4, 6, 8, 10, 12 and 24 h) were analyzed spectrophotometrically at 253 nm using PBS pH 7.4 solution as a blank.

## Optimization of DZ-loaded cubosomal dispersion formulae

Design^®^ expert 8 software (Stat-Ease, Inc., Minneapolis, MN) was used to suggest the optimized formulation based on the criteria of minimum PS, highest Zp (absolute value), with lowest percent of drug released after two hours (Q 2 h). The optimum DZ-loaded cubosomal dispersion having the maximum desirability value was elected for extra investigations. Morphological investigation of the optimized DZ-loaded cubosomal dispersion was further confirmed using Transmission electron microscopy (TEM) (Joel, Jem-2100, Tokyo, Japan) run at 100 kV. A droplet of the optimized cubosomal dispersion was located on a 200 mesh carbon-coated copper grid, and was stained with 1% sodium phosphotungstate solution before TEM investigation (Hashem et al., 2018).

## Preparation of the optimized DZ-loaded cubogel

DZ-loaded Cubogel was prepared by addition of Carbopol 934 (1% w/v) on the optimized DZ cubosomal dispersion under continuous stirring, few drops of triethanolamine were added to adjust pH to 7.4. The prepared cubogel was left overnight in the refrigerator till further investigations (Morsi et al., 2014; Shamma et al., 2019; Ahmed et al., 2020).

## *In-vitro* characterization of the optimized DZ-loaded cubogel

### pH determination

The prepared DZ-loaded cubogel was evaluated for pH determination using pH Meter (Jenway, type (3510), United Kingdom) (Acharya et al., 2019; Wadhwa et al., 2019).

### Rheological study

The viscosity of the optimized DZ-loaded cubogel was determined using a rotational viscometer of cone and plate structure (Model HBDV-I + CP, Spindle CPE-41, Middleboro, MA, USA) at 25 ± 2 °C. The measurements were performed at different shear speeds (1– 60 rpm). The average of two readings viscosity was calculated (Younes et al., 2018; Wadhwa et al., 2019).

### Transmission electron microscopy (TEM)

A droplet of the optimized DZ-loaded cubosomal dispersion and a small amount of cubogel were located on a 200 mesh carbon-coated copper grid, stained with 1% sodium phosphotungstate solution before TEM investigation (Hakeem et al., 2020).

### Differential scanning calorimetry (DSC)

This test aims to discover any possible change in the physical state of DZ following formulation of cubogel. DSC (DSC-60, Schimadzu, Kyoto, Japan) was carried out for pure DZ, F127, Carbopol 934 and the optimized DZ-loaded cubogel. Samples (5 mg) were sealed into aluminum pans under a nitrogen atmosphere flow (50 ml/min) and heated over a temperature range 30–450 °C at a constant rate of 10 °C/min (Hashem et al., 2018).

### Ex-vivo corneal permeation

The corneal permeation from the optimized cubogel compared to Trusopt^®^ eye drops were agreed by Research Ethics Committee, Faculty of Pharmacy, Cairo University (REC-FOPCU) (PI 2529). This test was carried out using excised corneal membranes extracted from anesthetizing male albino rabbits (weight 2–3 kg) following intramuscular injection of 35 mg/kg ketamine and 5 mg/kg xylazine. The rabbits were beheaded to remove the cornea and sclera, which were cleaned using PBS (pH7.4) and immediately displayed on the acceptor cell filled with 20 ml PBS (pH7.4) whereas the central transparent cornea was only exposed to the permeation medium. In the donor cell one ml of PBS (pH7.4) was mixed together with a certain amount of the optimized cubogel or Trusopt^®^ eye drops equivalent to 5 mg DZ. The permeation medium was thermostatically controlled at a temperature of 32.5 ° C ± 0.5 ° C under a rotation speed of 100 rpm.

Samples (0.5 ml) were taken from the acceptor cell using a tuberculin syringe at different time intervals; 0.5, 1, 2, 3, 4, 6, 8, 10, 12 and 24 h respectively, where aliquots (0.5 ml) were taken and renewed with fresh PBS (pH7.4). The gathered samples were filtered using 0.45 μm membrane filter. Samples were investigated using HPLC (Shimadzu, Tokyo, Japan) attached to Hypersil C18 column (150 × 4.6 mm, 5 μm) and UV detector at the predetermined *λ*_max_ (254 nm). The mobile phase consisted of 7% CH_3_CN and 93% of a solution containing 1% triethanol amine (Hakeem et al.) adjusted to pH 3.5 with ortho phosphoric acid (H_3_PO_4_.) The mobile phase was filtered through 0.45 µ membrane filter and sonicated for 10 min, running with a flow rate 1.2 ml/min (Maltese & Bucolo, 2002).

### Stability study

The physical stability should be carefully studied as investigation of only morphological features as a function of time could not give a real idea about the stability feature of the developed formulation. Therefore, the optimized DZ-loaded cubogel was subjected to stability testing as per as ICH guidelines (Nasr et al., 2015; Alharbi et al., 2020; Rajani et al., 2020) by keeping in refrigerator at a temperature (4 °C) in aluminum foil sealed glass vials. Characterization tests such as DC (%), PS (nm), PDI, Zp (−mV) and *in-vitro* release were reevaluated after suitable dilution to compare between fresh and stored cubogel formula respectively (Nasr et al., 2015).

Similarity factor ''ƒ2'' was determined to suggest whether there is difference between the release profiles of the optimized DZ-loaded fresh and stored cubogel by applying in the given equation (Elsayed & Sayed, 2017; Sayed et al., 2018):

f2=50·log{[1+(1/n)∑t=1n(Rt−Tt)2]−0.5·100}

*n*: the number of sample times of release. *R_t_* and *T_t_*_:_ the release of fresh and stored samples at time t. If ‘'ƒ2’' value lies between 50 and 100, this indicates that the two release profiles are similar (Sarfraz et al., 2006).

## *In-vivo* characterization of the optimized DZ-loaded cubogel

### Eye toxicity draize test

The draize rabbit test was carried out as to test the compatibility of the prepared cubogel formula when applied to mucous membranes regarding irritation and toxicity. Eye toxicity test was developed in 1940s, and was officially consented in the Organization for Economic Cooperation and Development (OECD) Guidelines for regulatory purposes of chemicals irritation (Draize et al., 1944).

Nine male albino New Zealand rabbits, weighing 2.5–3 kg were utilized in this test. Three groups (gp) of rabbits was made randomly, each having three rabbits as follows: rabbits of gp (I) received the market product Trusopt^®^ eye drops, those of gp (II) received the optimized cubogel and rabbits of gp (III) received iso propyl alcohol 90%.

The treatment was carried out in the right eye, left eye was left as control and took no treatment (saline solution). The animals should be kept in an isolated cage and received food and water over night before treatment. The right eye was visually examined after 72 h after the application of both optimized cubogel and Truspot^®^ eye drops then compared to group of rabbits received iso propyl alcohol 90%.

A score for erythema (redness) was given as shown (Bin-Jumah et al., 2020):

- Score 0, no response.
- Score 1, weak spread erythema.
- Score 2, weak with well definite erythema.
- Score 3, sufficient erythema.
- Score 4, severe erythema combined with edema.
- Score 5, very severe erythema combined with side effects.
- Score 6, showed irritation reaction.

### Histopathological examination

The biocompatibility of the optimized cubogel with corneal tissues was evaluated by histopathological examination of the eyeballs of three male albino rabbits (2.5–3.0 kg). One rabbit received one drop from isopropyl alcohol 95% (used as a positive control group), while one drop from normal saline was dripped into the eye of second rabbit (used as a negative control group) and finally the third rabbit received one drop of the optimized cubogel (Elsayed & Sayed, 2017; Maltese & Bucolo, 2002; Sayed et al., 2018).

Corneal sections were placed on glass slides, deparaffinized, stained by hematoxylin and eosin stain for evaluation under light electric microscope (DMS1000 B; Leica, Cambridge, UK) (Elsayed & Sayed, 2017; Sayed et al., 2018).

### Confocal laser scanning microscopy (CLSM)

This test was applied to image the corneal layers and evaluate the ability of the tested formulation to boost drug corneal permeation lacking any mechanical hurtful of the assessed tissues (Deng et al., 2020; Elsayed & Sayed, 2017; Sayed et al., 2018).

Observation of fluorescently-labeled cubosomal systems within the cornea was performed using Invert CLSM (LSM 710; Carl Zeiss, Jena, Germany). DZ in the optimized cubogel was substituted by 0.1% w/w Rhodamine B (RhB) to be imagined under CLSM. 100 µL was placed into both eyes of a male albino rabbit (2.5–3 kg) up to 6 h, and contrasted to (RhB) aqueous solution. After that, rabbits were anesthetized and decapitated (Salama & Shamma, 2015).

Clear corneas were gently eliminated, cleaned, kept in artificial tears and examined. Confocal images were managed and fixed using LSM software version 4.2 (Carl Zeiss MicroImaging, Jena, Germany) (Abdelbary et al., 2016a). Evaluation began from the outer corneal surface with the *z*-stack mode in the directions *xy* and *xz*. Surface by surface was imaged with 3 mm increases until the complete RhB color disappearance (Elsayed & Sayed, 2017; Sayed et al., 2018).

### Effect on intraocular pressure (IOP)

This study was approved by the committee of ‘Animal Care and Use’, Faculty of Pharmacy, Cairo University (PI 2529). A single-dose cross over design was used to study the pharmacological effect of the optimized DZ-loaded cubogel compared to Trusopt^®^ eye drops (market product) using six male healthy normotensive albino rabbits (2–3 kg). Treatments were applied topically into the upper quadrant of rabbit’s eye where in the first period, two groups of three rabbits received either Trusopt^®^ or optimized DZ-loaded cubogel. At different time intervals (0.5, 1, 2, 3, 4, 5, 6, 7, 8, 9, 10, 11 and 12 h) intra ocular pressure (IOP) was determined using Schiötz Tonometer (Rudolf Riester GmbH and Co. KG, Germany) and the mean values (±SD) of three replicates were calculated. After one-week washout period, the same procedure was repeated in cross over design. In all treatments, one eye is treated and the other is kept as a control. The percentage decrease in IOP was determined according to the following equation (Ammar et al., 2009):

$$\%\text{ Decrease in IOP }=\frac{{\text{IOP}}_{\text{Control eye}}–{\text{IOP}}_{\text{dosed eye}}}{{\text{IOP}}_{\text{Control eye}}}\times 100$$

The pharmacodynamics parameters detected were: maximum percentage decrease in IOP, time for maximum percentage decrease in IOP (*t*_max_), area under percentage decrease in IOP versus time curve (AUC_0–12_ h), and mean residence time (MRT). These parameters were determined using kinetica^®^ 2000 software.

### Effect of terminal gamma sterilization

Gamma sterilization was done in glass bottle with screw cap in the existence of dry ice to prevent any side effects that could happen as a result of high temperature caused by gamma irradiation which was achieved via Co irradiator at the dose rate of 1.774 kGy/h 66. Samples were irradiated at a dose 25 kGy in an Indian Gamma cell (Weyenberg et al., 2005).

The optimum cubogel following gamma irradiation was reevaluated for DC (%), PS (nm), PDI, Zp (−mV) as previously described before. Results were compared using SPSS^®^ program (IBM SPSS statistics, virgin 22), where similarity factor (*f*_2_) were used to differentiate the release profiles.

### Statistical analysis

Duos of groups were tested either by one-tailed Student *t*-test or multiple group assessment by one-way ANOVA and LSD using SPSS^®^ 22 to determine the significance of difference between measurements when the *p* values were less than .05.

All results are presented as mean values with their standard deviation (mean ± SD). ANOVA testing was also applied for IOP max decrease, AUC _0–12 h_ and MRT, also non-parametric Wilcoxon signed ranks test for *t*_max_ was applied to detect the significant difference between the different treatments in the *in-vivo* study.

## Results and discussion

### Preparation of DZ-loaded cubosomal dispersion

DZ-loaded cubosomal dispersions were effectively fabricated lacking the use of organic solvents to offer a safe ocular drug delivery. ‘Melt dispersion-emulsification’ method has the ability to form cubosomes with a homogenous and stable milky like dispersion against aggregation (Karami & Hamidi, 2016).

Preparation of cubosomal dispersions was built on the emulsification of the amphiphilic lipid (MO) being a good penetration enhancer together with high bioadhesive property (Ganem-Quintanar et al., 2000; Montis et al., 2015; Murgia et al., 2015) together with nonionic surfactant (F127) as stabilizing agent (Rizwan & Boyd, 2015). The lipid mixture was melted by the aid of thermostatically controlled magnetic stirring with different speed at 60 ± 2 °C. The use of Tween 80 aqueous solution as a dispersion medium (0.5% w/v) stabilized the final cubosomes to be used for ocular application (Azhari et al., 2016).

### Characterization of DZ-loaded cubosomal dispersion formulae

#### Drug content DC (%)

Table 2 shows results of DC of the different prepared DZ-loaded cubosomal dispersions, DC ranged from 90.50 ± 0.60 to 98.02 ± 0.42% which was within acceptable limits stated by the USP (85–115%) (AL-sakini & Maraie, 2019).

#### Particle size PS (nm) and polydispersity index (PDI)

**Figure 1.**
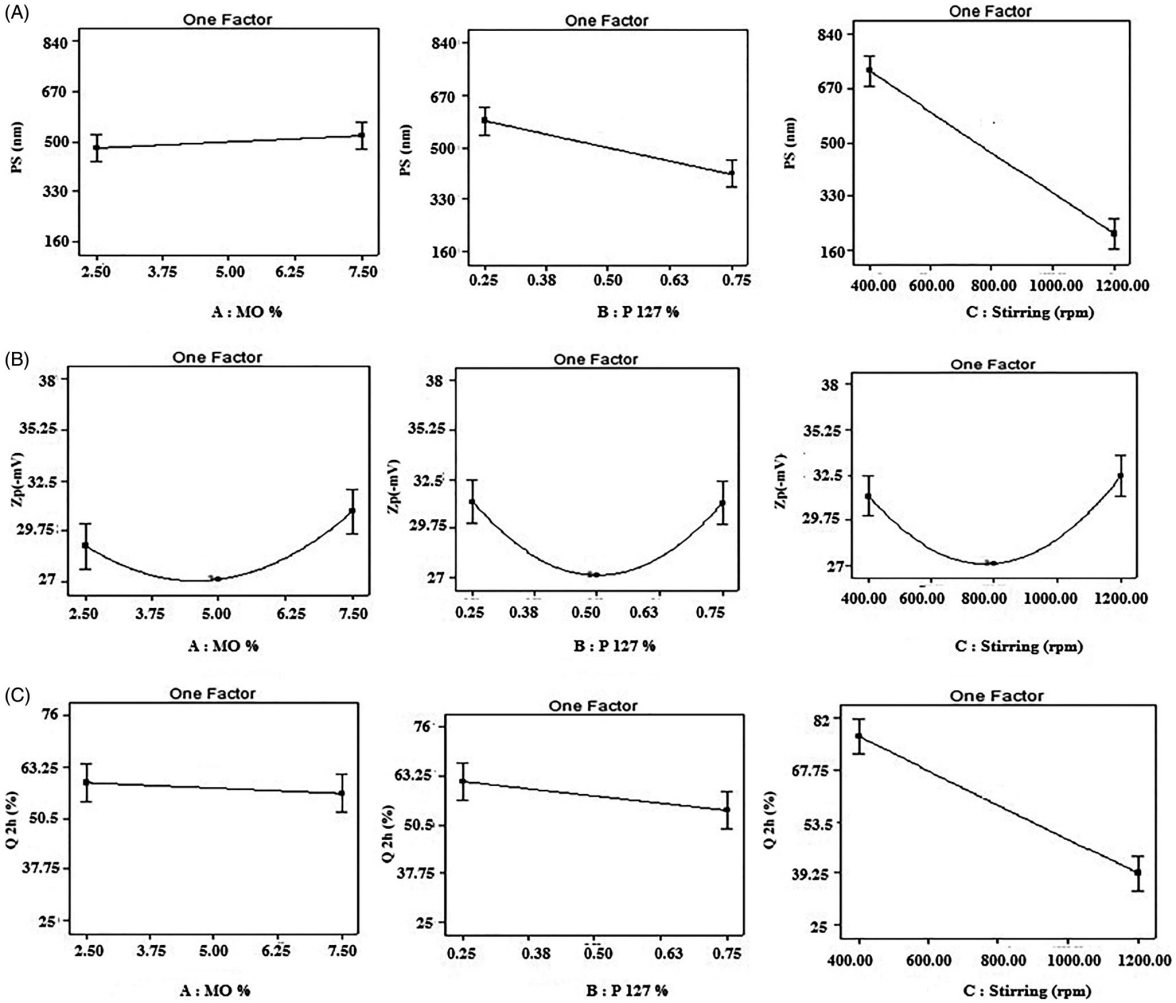
One Factor plots showing the effect of A: MO conc (% w/w), B: F127 conc (% w/w) and C: Speed of stirring (rpm) on: (A) PS (nm), (B) Zp (−mV), (C) Q 2 h (%).

Results of PS values of the different prepared DZ-loaded cubosomal dispersions were in the nanometric range; 161.55 ± 0.25 to 833.45 ± 269.55 nm as shown in Table 2 PDI values were found to be less than one with a range value of 0.192 ± 0.010 to 0.759 ± 0.241, showing suitable uniformity and high potential for corneal transportation (Huang et al., 2017). Values of PS of the prepared cubosomal dispersions were investigated using polynomial quadratic model with adequate precision of 19.55 and realistic difference between the predicted *R*^2^ (0.8797) and the adjusted R^2^ (0.9329). Figure 1(A) shows the effect of MO (A), F127 (B) and speed of magnetic stirring (C) on PS of the prepared cubosomal dispersions. It is clear from Figure 1(A) that both effects B and C are significant model terms (*p* = .0017 and *p* = .0001 respectively), while factor (A) shows a non-significant positive linear effect on PS (*p* = .3171).

Final equation of PS analysis in terms of coded factors was written as follows:

$$\text{PS }\left(\text{nm}\right)\;=\text{5}00.\text{87 }+\text{2}0.\text{8A }-\;\text{86}.\text{77 B }-\;\text{256}.\text{65C }+\text{55}.\text{35 BC }-\;\text{59}.{\text{88C}}^{\text{2}}$$

Concerning the concentration of F127 (B), a significant negative linear effect on PS was observed (*p* = .0017). Negative sign in equation indicated a significant inverse relation between PS and concentration of F127, where an increase in surfactant concentration resulted in a decrease in PS, F 127 being a nonionic surfactant reduces the surface tension and facilitating particle partition (Kancharla et al., 2020), also it has a high HLB value (HLB = 18) allowing it to be a good o/w emulsifier thereby stabilizing the aqueous phase and decreasing the particle size of the formed cubosomes (Kamel et al., 2009). Similar results were obtained from previous work (Abdelrahman et al., 2015; Salah et al., 2017), where a reduction of PS of the formed cubosomal dispersion was attained by increasing F127 concentration.

Regarding the speed of magnetic stirring (C), which had a significant negative linear effect on PS *(p* = .0001), where an increase in speed of magnetic stirrer from 400 to 1200 rpm decreased PS, as high magnetic stirring speed can form nanocubic vesicles with smaller PS, resulted from raising the shearing energy which break down large particles into smaller ones (Subara et al., 2018).

#### Zeta potential Zp (−mV)

Zeta potential values of the prepared DZ-loaded cubosomal dispersions were determined to predict the surface charge of the prepared nanoparticles to anticipate long term stability of the colloidal dispersion, high Zp values provide sufficient electric repulsion which in turn prevent particles’ aggregation (Pal et al., 2011).

Results of Zp as shown in Table 2 ranged from 27.15 ± 2.95 to 37.45 ± 1.0 (−mV), it is known that value at least equals to ± 30 mV was regularly essential to attain a logically stable dispersion (Thomas & Viswanad, 2012). Results of Zp were analyzed using polynomial quadratic model showing the predicted and adjusted *R*^2^ of 0.6938 and 0.8661 respectively, with adequate precision of 10.74.

A significant quadaric effect occurred (*p* < 0.05) with the following final equation:

$$\text{Zp }\left(-\text{mV}\right)\;=\;+\text{27}.\text{15}+0.\text{94 A}-0.0\text{44 B}+0.\text{62 C }+\text{2}.{\text{74 A}}^{\text{2}}+\text{4}.0{\text{8 B}}^{\text{2}}+\text{4}.{\text{7 C}}^{\text{2}}$$

Regarding factors of A, B and C had significant positive quadratic effect (*p* = .0035, *p* = .0003 and *p* = .0001, respectively).

As shown in Figure 1(B) increase (factor A) MO concentration lead to decrease in Zp until certain concentration (5% w/w), after that any further increase in MO concentration increase Zp. The high concentration of MO lead to high Zp, due to the existence of negative charge developed from the presence of free oleic acid and two OH groups of the glycerol moiety that confer to the negative polar head (Hundekar et al., 2014; Kulkarni et al., 2011; Mohyeldin et al., 2016), so that high negative charge of Zp indicated high repulsion between nanoparticles, which by turn means higher stability (Fouda et al., 2018).

While F127 concentration (factor B) when increase lead to decrease in Zp until concentration of 0.5 (% w/w) after which Zp increase with increase F127 concentration . F 127 has a stabilizing effect which provides not only effective electric repulsion preventing aggregation of cubosomal dispersion nanoparticles (Elnaggar et al., 2015), but also provided an electrostatic barrier between particles to prevent close particles to come in contact and thus keeping the dispersed particles in a stable form as confirmed in Figure 1(B) (Elnaggar et al., 2015; Gaballa et al., 2019).

Regarding the speed of magnetic stirring (factor C), when increase in speed lead to decrease Zp up to 800 rpm after which when increase speed lead to increase Zp as shown in Figure 1(B). It was previously reported that Zp increased with high stirring speed, owing to the inhibition of cubosomal dispersion nanoparticles agglomeration as a result from the agititation of the magnetic stirring which is responsible for stability and control of nanoparticales (Marinho et al., 2018; Subara et al., 2018).

#### *In-vitro* release (Q 2 h)

*In-vitro* release values of DZ from the different prepared cubosomal dispersions are shown in Table 2, values of Q 2 h ranged from 25.64 ± 0.33 to 75.67 ± 0.75%. Q 2 h values were analyzed using polynomial linear model found adequate precision of 12.52. The predicted *R*^2^ of 0.6808 and adjusted *R*^2^ of 0.8053. Response module of Q2h is significant (*p* = .0001), with final equation in terms of coded factors for Q 2 h:

Q2 h= +58.10 −1.34 A −3.74B −18.89 C

Concentration of MO factor (A) and F127 factor (B) had non-significant linear effect (*p* = .5990 and *p* = .1590) respectively on Q2 h, while speed of magnetic stirring factor (C) had a significant linear effect (*p* = .0001).

Increasing the stirring speed from 400 to 1200 rpm resulted in smaller PS cubosomal dispersions, with narrow pore size of the aqueous channels, consequently leading to a slower DZ release as a result of the limited diffusion of drug molecules incorporated in the aqueous channels (Anderson & Wennerstroem, 1990; Clogston et al., 2005; Abdelrahman et al., 2015), as shown in Figure 1(C).

## Optimization of the different prepared DZ-loaded cubosomal dispersion

**Figure 2.**
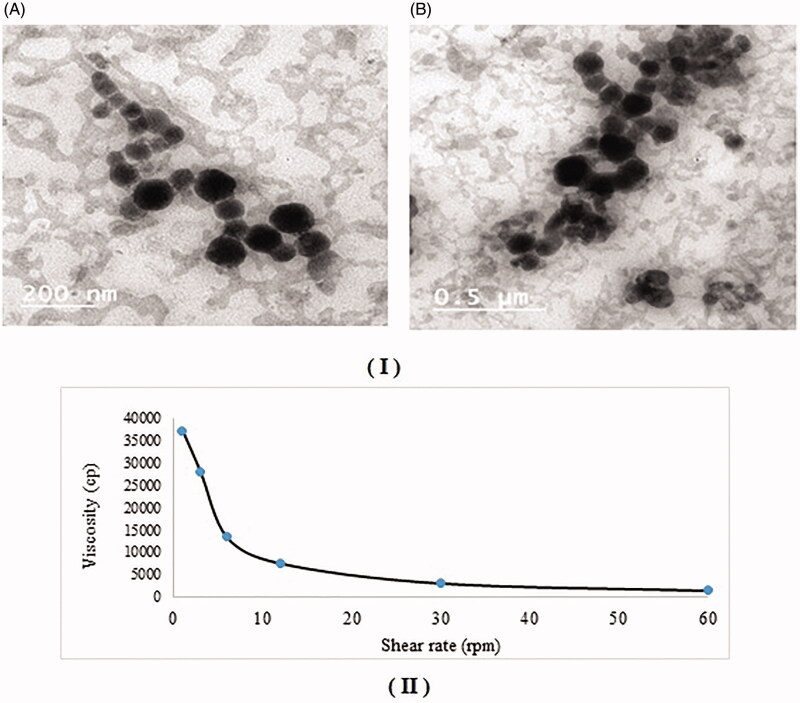
(I) Transmission Electron Micrographs (TEM) of: (A) Optimized DZ-loaded cubosomal dispersion, (B) Optimized DZ-loaded cubogel, (II) Rheological characterization of optimized DZ-loaded cubogel (a plot of viscosity versus shearing rate).

DZ-loaded cubosomal dispersion with desirability value of (0.933), was chosen as the optimum formula using Design expert^®^ software. This desirability value corresponds to DZ-loaded cubosomal dispersion composed of 6.13% w/w MO, 0.75% w/w F127 and prepared at stirring speed of (1200 rpm) respectively. Table 3 shows both theoretical and actual values of the different responses from the optimized formula, with a percent deviation below 20%. Figure 2(I-A) shows TEM of the optimized DZ-loaded cubosomal dispersion. It is clear from TEM micrographs that cubosomal particles are cubic in shape with uniform nano size confirming the results obtained in particle size study (Morsi et al., 2014).

**Table 3.** Theoretical and Actual values of the different responses for the optimized DZ-loaded cubosomal dispersion formula with prediction intervals and percent deviation.

Responses (Dependent Variables)	Theoretical values	Two sided 95% prediction interval		Actual values ± SD	% Deviation
		Low	High		
Y1: PS (nm)	162.288	161.5	833.48	153.3 ± 8.4	5.4
Y2: Zp (−mV)	37.4769	27.15	37.45	32 ± 3	14.4
Y3: Q2h (%)	34.8711	25.64	75.67	37.78 ± 1.3	−8.34

Preparation of the optimized DZ-loaded cubogel was done by addition of carbopol 934 (1% w/v) as previously discussed. The optimized DZ-loaded cubogel was further evaluated as follows:

## *In-vitro* characterization of the optimized DZ-loaded cubogel

### pH determination

pH is the most important parameter for ophthalmic formulation due to its effect on solubility and stability (Acharya et al., 2019; Wadhwa et al., 2019), at the same time to avoid irritation upon administration. The pH of the prepared cubogel was 6.97 ± 0.02.

### Rheological study

Viscosity play an important role in drug release and its bioavailability as it can affect spreadability and residence time at the application site (Salah et al., 2017). Figure 2(II) shows the viscosity of optimized cubogel which decrease when rpm increase (Younes et al., 2018).

### Transmission electron microscopy (TEM)

Figure 2(I-B) shows TEM of the optimized DZ-loaded cubogel had the same nano vesicular cubic shape with uniform nano size compared to TEM of the optimized DZ-loaded cubosomal dispersion (Hakeem et al., 2020).

### Differential scanning calorimetry (DSC)

**Figure 3.**
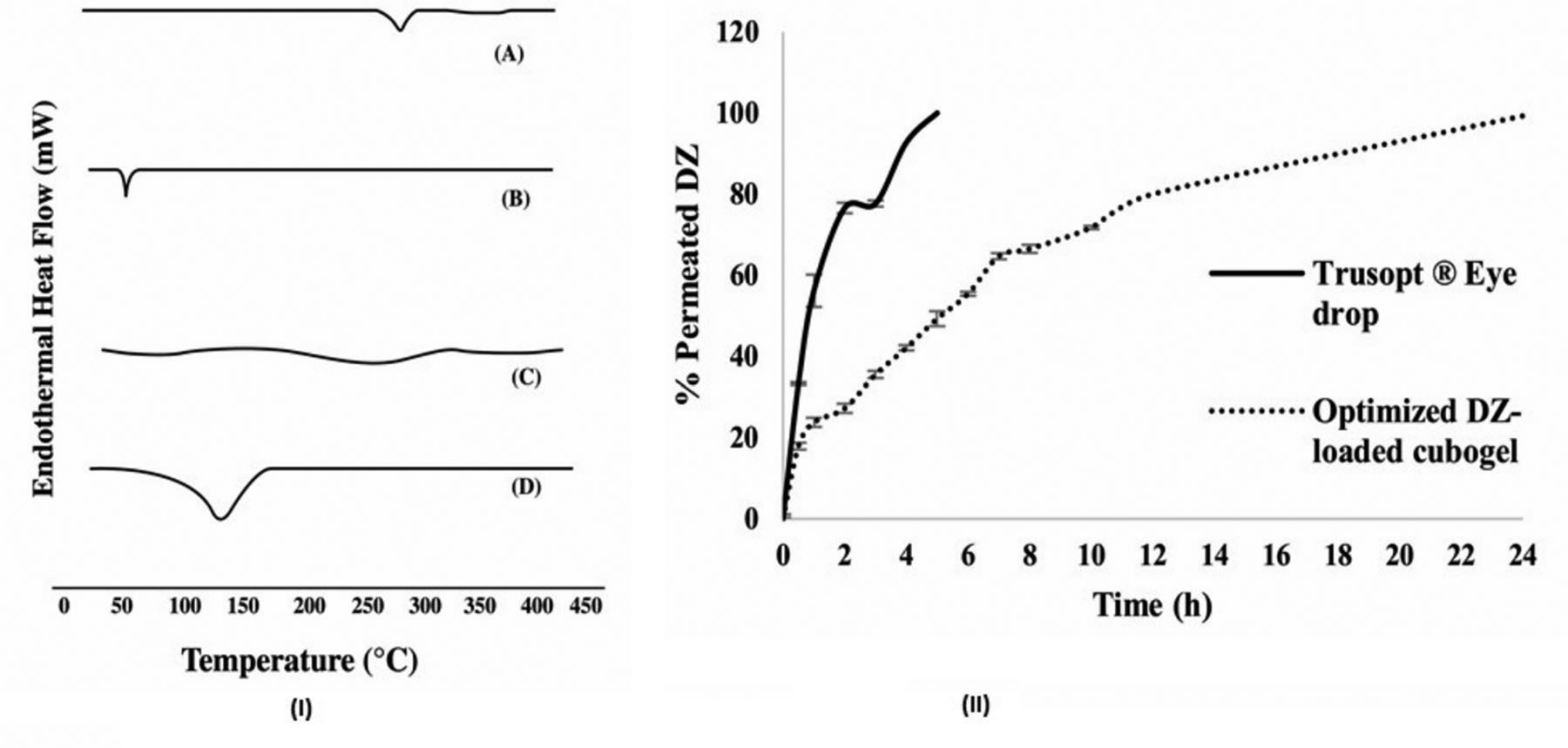
(I) DSC  thermograms of: (A) Pure DZ, (B) F127, (C) Carbopol^®^ 934 and (D) DZ-loaded optimized cubogel, (II) *Ex-vivo* corneal permeation of the optimized DZ-loaded cubogel and the market product Trusopt ^®^ eye drops.

Figure 3(I) displays the DSC thermograms of pure DZ, F127, Carbopol^®^ 934 and the optimized DZ-loaded cubogel. DSC thermogram of pure DZ showed an endothermic (277.6 °C), consequent to the melting point of crystalline form of the drug DZ. Figure 3(I-A) (Nikouei et al., 2013), while F127 revealed an endothermic peak consequent to its melting point at 56.3 °C Figure 3(I-B) Two baseline endotherms were shown between 50 °C and 100 °C due to the evaporation of Carbopol^®^ 934 moisture with increase in temperature Figure 3(I-C) (Amal El Sayeh & El Khatib, 2014). The complete disappearance of the sharp endothermic peak of DZ-loaded cubogel Figure 3 (I-D) revealed that DZ was incorporated into the cubosomal nanoparticles in its amorphous state (Younes et al., 2018).

### *Ex-vivo* permeation

As shown in Figure 3(II) the *ex-vivo* permeation experiment revealed that the optimized cubogel was more capable of increasing the corneal penetration with a significant difference in prolonging the drug retention time in the eye than the market eye drops Trusopt ^®^ at Q 2 h and Q 8 h (*p* = .024 and *p* = .001 respectively).

The optimized cubogel being cubic in shape with strong bioadhesive properties will enhance the corneal penetration and control drug release from the prepared cubogel formula as a result of small pore size (153.3 ± 8.4 nm) allowing great corneal transportation, also the presence of lipid phase (MO) which is the main precursor of cubosomal formation responsible of mucoadhesiveness of DZ-loaded cubogel with the cornea (Nagayasu et al., 1999; Clogston et al., 2005; Azhari et al., 2016). Also carbopol decreased rate of the DZ released from the optimized cubogel due to the increased viscosity (Amal El Sayeh & El Khatib, 2014).

### Stability study

Table 4 shows the *in-vitro* evaluation tests; DC (%), PS (nm), PDI, Zp (−mV) and *in-vitro* release of the optimized DZ-loaded cubogel at different storage time intervals (15, 30, 60 and 90 days) at 4 ± 3 °C compared to results of the freshly prepared formula. A non-significant difference was obtained with *p* values of .494, .754 and .208 for DC, PS and Zp respectively, after 90 days. Similarity factor (*f*_2_) between *in-vitro* release of the freshly prepared cubogel and after 90 days of storage was calculated and was found to be equal to 69.97 which is within the similarity range (50–100) (Abdelbary et al., 2016b). Comparison of DZ release at Q 2 h (*p* = .816) and Q 8 h *(p* = .267) between fresh samples and after 90 days of storage at 4 ± 3 °C showed a non-significant difference, confirming the stability of the lipid phase MO at the storage temperature (Chong et al., (2015)). Results of PDI (PDI < 1) confirmed also suitable homogeneity and high potential to corneal permeation (Huang et al., 2017).

**Table 4.** Effect of storage conditions on: DC (%), PS (nm), PDI, Zp (−mV) and *in-vitro* release at Q 2 h and Q 8 h of the optimum DZ-loaded cubogel formula at 4 ± 3 °C at different time intervals (0, 15, 30, 60 and 90 days).

Evaluation tests at different storage time intervals						
	DC	PS	PDI	*Zp*	Q 2h	Q 8h
Storage intervals	(%± SD)	(nm ± SD)	(± SD)	(-mV ± SD)	( % ± SD)	( % ± SD)
Fresh	96.71 ± 0.98	216.90 ± 11.13	0.328 ± 0.007	47.55 ± 0.15	27.27 ± 0.86	68.00 ± 2.00
15 days	96.30 ± 0.52	220.50 ± 19.7	0.260 ± 0.133	45.50 ± 1.20	27.59 ± 0.97	68.75 ± 2.25
30 days	95.60 ± 0.90	196.35 ± 29.45	0.474 ± 0.025	48.30 ± 7.20	27.97 ± 1.13	69.00 ± 1.00
60 days	95.75 ± 0.26	212.35 ± 33.95	0.351 ± 0.055	49.50 ± 0.90	28.54 ± 1.05	70.25 ± 0.75
90 days	95.65 ± 2.80	209.45 ± 38.45	0.456 ± 0.053	51.35 ± 2.35	28.20 ± 2.40	70.50 ± 0.50

## *In-vivo* characterization of the optimized DZ-loaded cubogel

### Eye irritancy draize test

**Figure 4.**
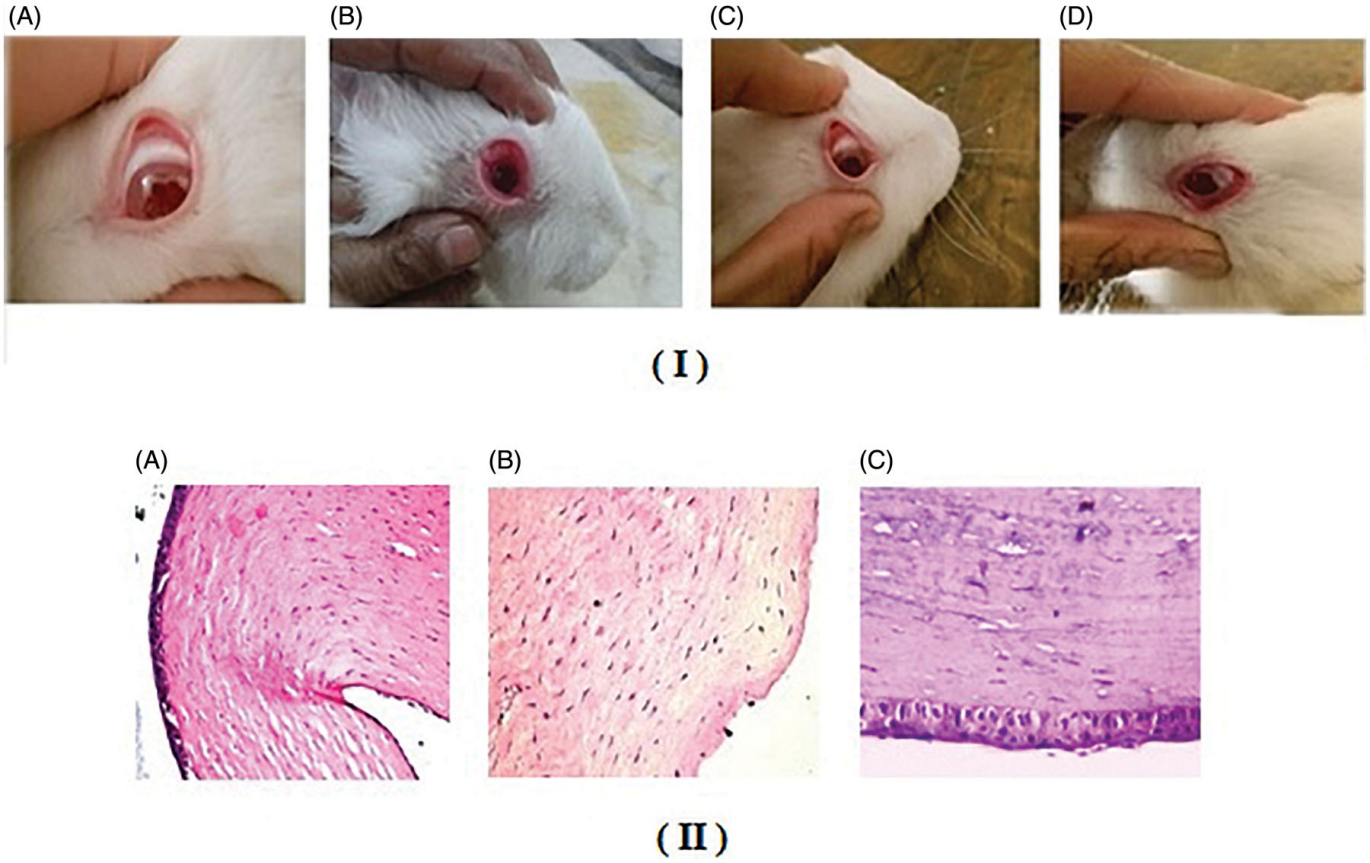
(I) *In-vivo* eye irritancy study ‘Draize test’ showing photos of the eyes of the rabbits after 72 h; (A) Control; (B) Isopropyl 90 %; (C) Trusopt^®^ eye drops and (D) Optimized DZ-loaded cubogel, (II) Photomicrographs showing histopathological sections of rabbit eye treated with (A) Normal saline, (B) Isopropyl alcohol 95% and (C) Optimized DZ-loaded cubogel.

Figure 4(I - (A–D)). shows the results of ocular irritancy testing. It is clear that the tested optimized cubogel did not confirm any symptom of redness, inflammation after 72 h of treatment compared to control eye. Therefore, it could be concluded that the optimized cubogel was nonirritant following topical application into the eye. During the 72 h, no irritation marks were observed (scored 0) as shown in Figure 4(I–D).

Rabbits received iso propyl 90% which showed irritation/reaction like conjunctival corneal edema and/or hyperemia through direct optical examination using a slit lamp and scored according to draize test score 6 as shown in Figure 4(I–B) (Di Colo et al., 2001).

### Histopathological examination

Histopathological test is essential to estimate the biocompatibility of the applied excipients used in the tested formula with the corneal tissue. Normal ocular structure in the negative control as shown in Figure 4(II-A), while Figure 4(II-B) shows destruction of epithelial cells as a result of isopropyl 90% acting as positive control. Optimized cubogel showed good biocompatibility as carrier for ocular drug release as no histological insufficiency or inflammation was detected as shown in Figure 4(II-C) (Huang et al., 2017). Therefore the optimized DZ-loaded cubogel composed of biodegradable and biocompatible materials was classified as GRAS (generally recognized as safe) as per FDA guides (Rao et al., 2018).

### Confocal laser scanning microscopy (CLSM)

**Figure 5.**
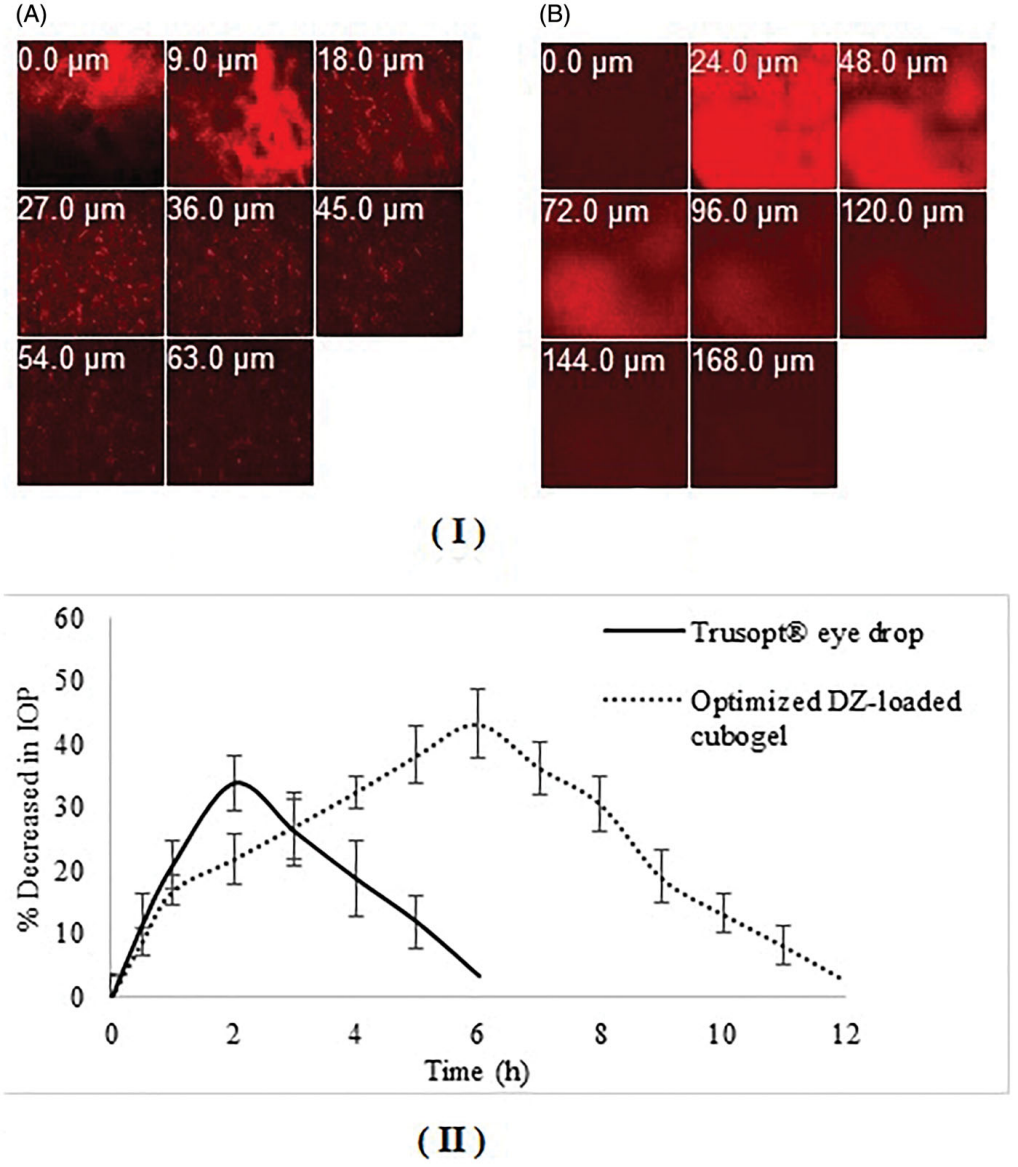
(I) Confocal laser scanning micrographs of the rabbit corneas after instillation of: (A) RhB-loaded aqueous solution (B) RhB-loaded optimized DZ-loaded cubogel, (II) Average percentage decrease in IOP (± SD) after administration of the market eye drops Trusopt ^®^ and the optimized DZ-loaded cubogel.

Confocal laser scanning microscopy was used to evaluate the capacity of the optimized cubogel to improve the corneal permeation of DZ, through observing the transcorneal behavior of RhB dye, by tracking its fluorescence inside the cornea Figure 5(I) shows that the penetration of RhB solution alone was recorded at 63 µm, while the optimized cubogel improved the penetration of RhB up to 168 µm, therefore CLSM is considered as quantitative analysis test for absolute dye penetration. Our finding was in harmony to our previously published work, where RhB permeated to a depth of 114 μm from the optimum ocular proniosomal gel (Sayed et al., 2020).

### Effect on intraocular pressure (IOP)

Effect of the optimized DZ-loaded cubogel and Trusopt^®^ eye drops on percent decrease of IOP of rabbits are shown in Figure 5(II). Results of pharmacodynamics parameters of the optimized cubogel and market eye drops Trusopt^®^ are given in Table 5.

**Table 5.** Pharmacodynamic parameters following IOP measurement of maximum decrease in IOP (%), Tmax (h), Mean residence time MRT (h) and area under the curve AUC 0–12 (mg.h/ ml) of the market eye drops Trusopt ^®^ compared to optimum DZ-loaded cubogel formula.

Pharmacodynamic parameters	Eye drop Trusopt^®^	Optimized DZ-loaded cubogel
Maximum decrease in IOP (%) ± SD	33.76 ± 3.81	43.18 ± 4.51
*T*_max_ (h) ± SD	2 ± 0	6 ± 0
MRT (h) ± SD	2.80 ± 0.29	5.88 ± 0.34
AUC _0–12_ (mg.h/ ml) ± SD	115.40 ± 21.42	288.42 ± 23.48

A higher significant (*p* = .033) decrease in IOP 43.18% at *t*_max_ 6 h was obtained from the optimized cubogel compared to Trusopt^®^ eye drops (33.76% at *t*_max_ 2 h). Regarding the mean MRT (5.88 h ± 0.34 and 2.80 h ± 0.29) for cubogel and Trusopt^®^ eye drops respectively, where a significant difference (*p* < .05) occurred between them. The increased MRT of the optimized cubogel is an evidence of the sustainment of drug release from the cubogel compared to the market eye drops which confirmed high ocular contact time (Chandraprakash et al., 1990; Ruckmani et al., 2010) as shown in Table 5.

Pharmacodynamic parameters of the optimized cubogel compared to Trusopt^®^ eye drops are shown in Table 5. It is clear that there is a significant increase (*p* < .05) in the bioavailability of the drug from the optimized formula more than Trusopt^®^ eye drops, where AUC _0–12 h_ equals to 288.42 h ± 23.45 and 115.40 h ± 21.42 respectively. About 2.5-fold improvement in the therapeutic efficacy could be resulted from the enhancement in ocular contact time with strong bioadhesive properties resulting in higher corneal permeation and penetration. The control of drug release from the optimum cubogel was due to its small pore size (153.3 ± 8.4 nm), allowing a sustained potential to transport throughout the cornea together with the presence of the main lipid phase (MO) having higher viscosity responsible of mucoadhesiveness of DZ-loaded cubogel and prolonged release inside the cornea (Azhari et al., 2016; Clogston et al., 2005; Nagayasu et al., 1999). In addition, Tween 80 acts as permeation enhancer allows more elasticity to the nanovesicles to pass easily through the corneal barrier (Shamma & Elsayed, 2013).

### Effect of gamma sterilization on the optimized DZ-loaded cubogel

Sterilization is very important for ocular dosage form to bypass co-infecting the patient with dangerous microorganisms present in the preparation. Terminal sterilization has minimal hazardous effect than aseptic sterilization due to the lack of any microorganisms in the final product (Masson et al., 1997).

Results of DC (%), PS (nm) and *Zp* (−mV) of the optimized DZ-loaded formula as shown in Table 6 revealed a non-significant difference in the values of DC (%), PS (nm) and *Zp* (−mV) before and after gamma sterilization (*p* values of .252, .891 and .284) respectively, while PDI showing good homogeneity (PDI <1) and high potential to corneal transportation (Huang et al., 2017).

**Table 6.** Effect of sterilization on: DC (%), PS (nm), ZP (−mV) and *in vitro* release at Q 2 h and Q 8 h of the optimum DZ-loaded cubogel formula.

Parameters	Before sterilization	After sterilization	*p* Value
DC (%) ± SD	95.38 ± 0.67	94.31 ± 1.20	.252
PS (nm) ± SD	190.15 ± 35.55	186.60 ± 22.5	.891
Zp (−mV) ± SD	48.15 ± 1.55	46.95 ± 0.65	.284
Q 2h (%) ± SD	26.03 ± 1.13	27.54 ± 1.45	.227
Q 8h (%) ± SD	68.25 ± 0.75	69.00 ± 1.00	.357

*In-vitro* release profiles of DZ from the optimized cubogel, exhibited a non-significant variance before and after gamma sterilization at Q 2 h and Q8 h (*p* = .227 and .3) respectively. Similarity factor (*f*_2_) before and after sterilization was computed and was equal to 88.777 which is within the similarity range (50–100) (Abdelbary et al., 2016b).

## Conclusion

Cubosomes were successfully fabricated and statistically optimized to obtain the most favorable physicochemical characteristics. Moreover, a significantly sustained *ex-vivo* corneal permeation was attained from the optimized cubogel compared to the market eye drops product (Trusopt^®^. Results revealed that the optimized cubogel was considerably safe, stable and competent to corneal delivery as assured by Draize and histological examination. CLSM showed a deeper penetration of more than 2.5-fold relative to aqueous Rhodamine solution. Gamma sterilization revealed a non-significant change in *in-vitro* cubogel characterization tests, suggesting that DZ-loaded cubogel could be treated as favorable ocular nano-delivery system in the healing of glaucoma.
